# Deep Learning–based Segmentation of Computed Tomography Scans Predicts Disease Progression and Mortality in Idiopathic Pulmonary Fibrosis

**DOI:** 10.1164/rccm.202311-2185OC

**Published:** 2024-03-07

**Authors:** Muhunthan Thillai, Justin M. Oldham, Alessandro Ruggiero, Fahdi Kanavati, Tom McLellan, Gauri Saini, Simon R. Johnson, Francois-Xavier Ble, Adnan Azim, Kristoffer Ostridge, Adam Platt, Maria Belvisi, Toby M. Maher, Philip L. Molyneaux

**Affiliations:** ^1^ Royal Papworth Hospital, Cambridge, United Kingdom;; ^2^ Qureight Ltd., Cambridge, United Kingdom;; ^3^ Division of Pulmonary and Critical Care Medicine, University of Michigan, Ann Arbor, Michigan;; ^4^ National Heart and Lung Institute, Imperial College London, London, United Kingdom;; ^5^ Translational Medical Sciences, National Institute for Health and Care Research Biomedical Research Centre and Biodiscovery Institute, University of Nottingham, Nottingham, United Kingdom;; ^6^ Translational Science and Experimental Medicine, Research and Early Development, Respiratory and Immunology, BioPharmaceuticals R&D, AstraZeneca, Cambridge, United Kingdom;; ^7^ Translational Science and Experimental Medicine,; ^8^ Research and Early Development, Respiratory and Immunology, BioPharmaceuticals R&D, AstraZeneca, Gothenburg, Sweden;; ^9^ Faculty of Medicine, University of Southampton, Southampton, United Kingdom;; ^10^ Keck School of Medicine, University of Southern California, Los Angeles, California; and; ^11^ Royal Brompton and Harefield Hospital, Guy’s and St. Thomas’ NHS Foundation Trust, London, United Kingdom

**Keywords:** IPF, machine learning

## Abstract

**Rationale:**

Despite evidence demonstrating a prognostic role for computed tomography (CT) scans in idiopathic pulmonary fibrosis (IPF), image-based biomarkers are not routinely used in clinical practice or trials.

**Objectives:**

To develop automated imaging biomarkers using deep learning–based segmentation of CT scans.

**Methods:**

We developed segmentation processes for four anatomical biomarkers, which were applied to a unique cohort of treatment-naive patients with IPF enrolled in the PROFILE (Prospective Observation of Fibrosis in the Lung Clinical Endpoints) study and tested against a further United Kingdom cohort. The relationships among CT biomarkers, lung function, disease progression, and mortality were assessed.

**Measurements and Main Results:**

Data from 446 PROFILE patients were analyzed. Median follow-up duration was 39.1 months (interquartile range, 18.1–66.4 mo), with a cumulative incidence of death of 277 (62.1%) over 5 years. Segmentation was successful on 97.8% of all scans, across multiple imaging vendors, at slice thicknesses of 0.5–5 mm. Of four segmentations, lung volume showed the strongest correlation with FVC (*r* = 0.82; *P* < 0.001). Lung, vascular, and fibrosis volumes were consistently associated across cohorts with differential 5-year survival, which persisted after adjustment for baseline gender, age, and physiology score. Lower lung volume (hazard ratio [HR], 0.98 [95% confidence interval (CI), 0.96–0.99]; *P* = 0.001), increased vascular volume (HR, 1.30 [95% CI, 1.12–1.51]; *P* = 0.001), and increased fibrosis volume (HR, 1.17 [95% CI, 1.12–1.22]; *P* < 0.001) were associated with reduced 2-year progression-free survival in the pooled PROFILE cohort. Longitudinally, decreasing lung volume (HR, 3.41 [95% CI, 1.36–8.54]; *P* = 0.009) and increasing fibrosis volume (HR, 2.23 [95% CI, 1.22–4.08]; *P* = 0.009) were associated with differential survival.

**Conclusions:**

Automated models can rapidly segment IPF CT scans, providing prognostic near and long-term information, which could be used in routine clinical practice or as key trial endpoints.

**At a Glance Commentary**

**Scientific Knowledge on the Subject**

A number of imaging tools have been developed to analyze computed tomography scans in idiopathic pulmonary fibrosis, but image-based biomarkers are still not routinely used in hospital practice or in clinical trials.

**What This Study Adds to the Field**

Deep learning was used to create anatomical segmentation models, which were applied to a unique set of treatment-naive patients from the PROFILE (Prospective Observation of Fibrosis in the Lung Clinical Endpoints) study and further validated against 195 real-world patients with idiopathic pulmonary fibrosis. These models can rapidly segment computed tomography scans and provide prognostic near- and long-term information, which could be used in routine clinical practice or as key trial endpoints.

Idiopathic pulmonary fibrosis (IPF) is a progressive and ultimately fatal condition for which there is no cure (1). Antifibrotic medications slow, but do not prevent, disease progression (2). In clinical practice, monitoring of IPF progression is performed using serial measurements of lung physiology, including FVC and Dl_CO_ (3–5). Measurement variability often makes the interpretation of short-term lung function trends difficult (6). Furthermore, many patients struggle to perform lung function because of fatigue or cough, and comparison of results obtained at different institutions is frequently challenging (7).

Thoracic computed tomography (CT) is the modality of choice for diagnosing individuals with suspected IPF and has largely supplanted lung biopsy, which carries a significant risk of morbidity and mortality in patients with lung fibrosis (8). Various CT features of fibrosis are predictive of prognosis when measured both at baseline and longitudinally (9–11). However, visual assessment and scoring of CT patterns is time consuming, subjective, and associated with high interobserver variability (12). Manual segmentation of CT scans into individual components (e.g., airways) is also possible but requires both expert radiology training and significant time to segment each CT scan. Although computer-assisted approaches for classifying and quantifying CT patterns of fibrosis have been available for nearly three decades, none is routinely used in either clinical practice or as an endpoint in trials (13). To be used as an effective biomarker, automated assessment of CT imaging needs to be agnostic to CT equipment, tolerant of technical differences between scans, reproducible, rapid to perform, and readily interpretable. As with other biomarkers, imaging algorithms require validation in prospective cohorts so that the relationship between change in any given imaging parameter and outcomes of importance to patients (disease worsening, death, etc.) can be clearly defined.

We sought to develop CT-based imaging biomarkers by using high-throughput segmentation consisting of four different anatomical models identified using deep learning methods. These models were applied to a unique cohort of treatment-naive patients with IPF enrolled in the PROFILE (Prospective Observation of Fibrosis in the Lung Clinical Endpoints) study, a multicenter prospective study of IPF conducted in the United Kingdom. Although a small number of patients in PROFILE were treated toward the end of the observational study, they were treatment naive at the point of data capture (as it was before the widespread use of antifibrotics). The relationships between CT imaging biomarkers and lung function, disease progression, and mortality were assessed at baseline and longitudinally.

## Methods

Study approval was obtained from ethics committees (10/H0402/2, 10/H0720/12). Incident IPF cases were recruited into PROFILE (14) after informed consent was obtained. Diagnosis for all patients with IPF occurred in multidisciplinary meetings, with clinical input from both pulmonologists and thoracic radiologists. Patients were assessed at baseline; at 1, 3, 6, and 12 months; and then annually for 3 years. Survival status was assessed on June 3, 2020. Thoracic CT scans were captured as part of routine care before enrollment and during follow-up. Pulmonary function testing (PFT) closest to CT acquisition (within 180 d) was used to ascertain baseline lung function relative to CT. The cohort was stratified into equally sized discovery and validation cohorts on the basis of the date of recruitment. Findings were also tested in an independent validation cohort of 195 patients with IPF from Royal Papworth Hospital (Cambridge cohort). These patients had CT scans analyzed at baseline (CT performed closest to the time of multidisciplinary diagnosis), and follow-up vital status was acquired by review of the medical record. Ethical approval was given by the hospital research and development committee (reference S02467).

Fibrosis and airway models were trained using supervised approach with CT scans as inputs and ground-truth segmentations as outputs. Ground-truth labeling for both models was bootstrapped by manual segmentation masks performed by two independent radiologists, each with more than 10 years’ IPF reporting experience. Airways were segmented from the trachea up to the last visible generation of distal airways. Fibrosis (areas of reticulation and honeycombing) were segmented in both lungs. Initial segmentation masks were used to train a first model, which was then applied to a new image set manually corrected by the radiologists. Intermediate models were iteratively retrained after 20–30 manual corrections were performed. Fibrosis and airway models were based on three-dimensional convolutional neural networks with UNet architecture consisting of encoder and decoder networks with skip connections. For fibrosis, images were resampled to 1.4-mm resolution and masked with lung segmentations obtained from an open-source model (15) also used for lung volume segmentation. To focus on intrapulmonary airways (up to the seventh generation), trachea and proximal main bronchi were removed by lung mask segmentation multiplication. Intrapulmonary vessels were segmented by applying the Frangi enhancement filter (16). A full description of the training and testing CT scans, detailed model development, and segmentation methods is provided in the online supplement.

Univariable and multivariable Cox proportional-hazards regression adjusted for baseline gender, age, and physiology (GAP) stage (13) at the time of CT was used to assess the primary endpoint of five-year overall survival, defined as the time from CT to death of any cause or censoring at 60 months or before if lost to follow-up. The PROFILE discovery and validation cohorts were then pooled and secondary analyses performed. Pearson correlation was used to estimate correlations between CT and baseline PFT measures. Two-year progression-free survival, defined as the time from CT to death, ⩾10% relative FVC decline, or censoring was assessed using a multivariable Cox proportional-hazards regression model adjusted for baseline GAP stage.

Annual change in CT features was then assessed using linear mixed-effects regression in a subset of individuals who underwent repeat CT 6–24 months after baseline CT (*n* = 134). This model included an exchangeable correlation structure and a random slope term, which produced the best model fit. Using simple linear regression, we then estimated patient-specific annualized changes in CT biomarkers and, using these values, categorized annual change in each CT biomarker as stable or decreasing versus increasing. The association between categorical change and subsequent two-year survival, defined as the time from second CT to death or censoring, was assessed using Cox proportional-hazards regression. Survival between categorical change groups was compared using a log-rank test and plotted using the Kaplan-Meier estimator.

## Results

### PROFILE Data Clinical and Demographic Analysis

Of 628 PROFILE participants, 426 met inclusion criteria (i.e., had at least a baseline CT scan, baseline PFT, and PFT values within 180 days of CT scan). CT segmentation resulted in approximately 7,800 segmentations derived from the four different models (fibrosis, airway, vascular, and lung volumes), with a success rate of 99.3%. The discovery and validation cohorts were well balanced in terms of age, sex, smoking history, lung function, and outcomes (Table 1). Forty-six percent (*n* = 97) of patients died within three years of baseline CT in the discovery cohort and 44% (*n* = 93) in the validation cohort. Nearly half of each cohort experienced IPF progression within two years (i.e., 24-mo progression).

**Table 1.** Baseline Characteristics and Outcomes for the PROFILE and Cambridge Cohorts

Characteristic	PROFILE Discovery (*n* = 223)*	PROFILE Validation (*n* = 223)^†^	Cambridge Validation (*n* = 195)^‡^
Age, yr, mean (SD)	69.4 (8.2)	70.8 (8.2)	72.6 (7.7)
Male sex, *n* (%)	179 (80.3)	170 (76.2)	142 (85.0)
Ever-smoker, *n* (%)	163 (73.0)	142 (63.7)	153 (78.5)
Pulmonary function, mean (SD)			
FVC% predicted	75.5 (18.9)	79.3 (18.9)	77.2 (15.5)
Dl_CO_% predicted	43.5 (15.7)	44.7 (14.6)	50.1 (14.4)
Lung volume, L, mean (SD)	4.02 (1.13)	4.00 (1.01)	3.97 (1.12)
Airway volume, L, mean (SD)	0.02 (0.01)	0.02 (0.01)	0.07 (0.03)
Vascular volume, L, mean (SD)	0.27 (0.10)	0.25 (0.09)	0.23 (0.08)
Fibrosis volume, L, mean (SD)	0.63 (0.33)	0.54 (0.26)	0.57 (0.26)
Outcomes			
24-mo progression	116 (52.0)	101 (45.3)	NA
60-mo mortality	150 (67.3)	127 (57.0)	138 (70.8)

*Definition of abbreviation*: PROFILE = Prospective Observation of Fibrosis in the Lung Clinical Endpoints.

* Number missing: FVC, *n* = 2; Dl_CO_, *n* = 11.

^†^ Number missing: FVC, *n* = 1; Dl_CO_, *n* = 8.

^‡^ Number missing: FVC, *n* = 28; Dl_CO_, *n* = 28.

### Model Training Results and CT Correlation with Lung Function

The performance of the fibrosis and airways models was validated using the Dice score coefficient, which is a measure of overlap widely used to assess segmentation performance against the ground truth (here represented by the testing dataset consisting of segmentations performed by the two independent radiologists and excluded from the training dataset) (17). With respect to the testing dataset, the airways segmentation model achieved a Dice score 0.93 ± 0.05. The fibrosis segmentation model achieved a Dice score of 0.90 ± 0.11. There was no correlation between Dice score and any of image attributes, such as scanner manufacturer, convolution kernel, or slice thickness.

**Figure 1.**
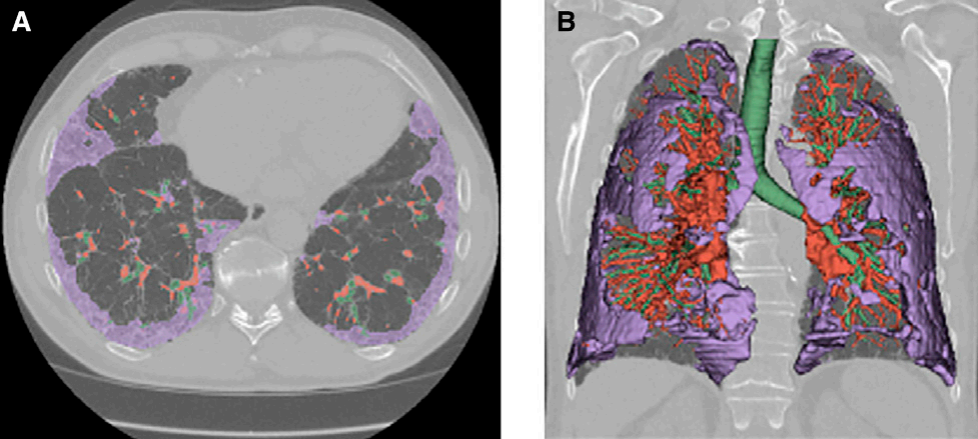
(*A* and *B*) Axial (*A*) and three-dimensional rendering (*B*) of an example high-resolution computed tomography scan that has been segmented to show areas of fibrosis (purple), blood vessels (red), and airways (green) within a lung mask.

**Figure 2.**
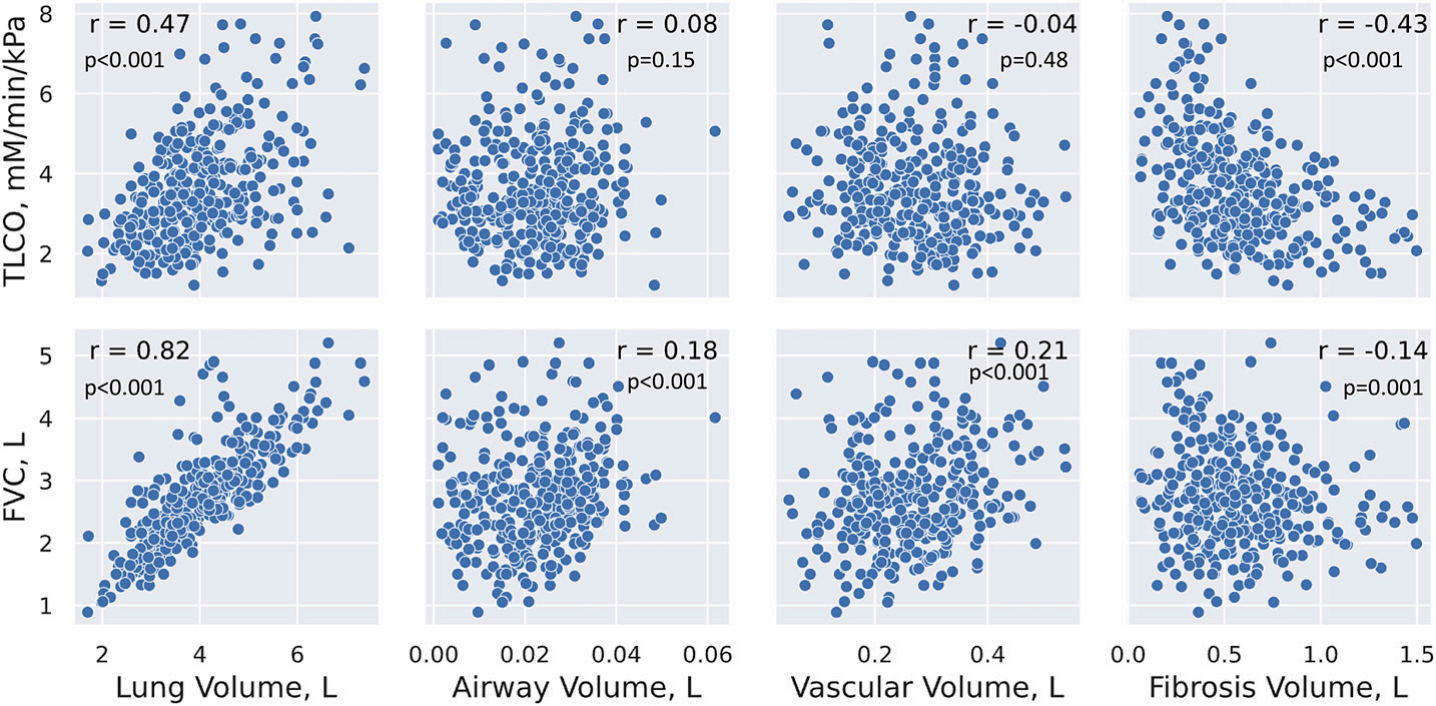
Scatterplots displaying relationships between pulmonary function testing (PFT) measures and high-resolution computed tomography (HRCT) segmentations. All PFT measurements were taken within 180 days of HRCT scans. TLCO = transfer capacity of the lung for carbon monoxide.

Baseline CT scans from PROFILE were each segmented into lung, airways, vascular, and fibrosis (Figure 1). Of the four imaging segmentations, lung volume showed the strongest correlation with FVC (*r* = 0.82; *P* < 0.001). No strong correlations were seen between imaging outputs and any other physiological variable (Figure 2).

### Survival Analysis

**Figure 3.**
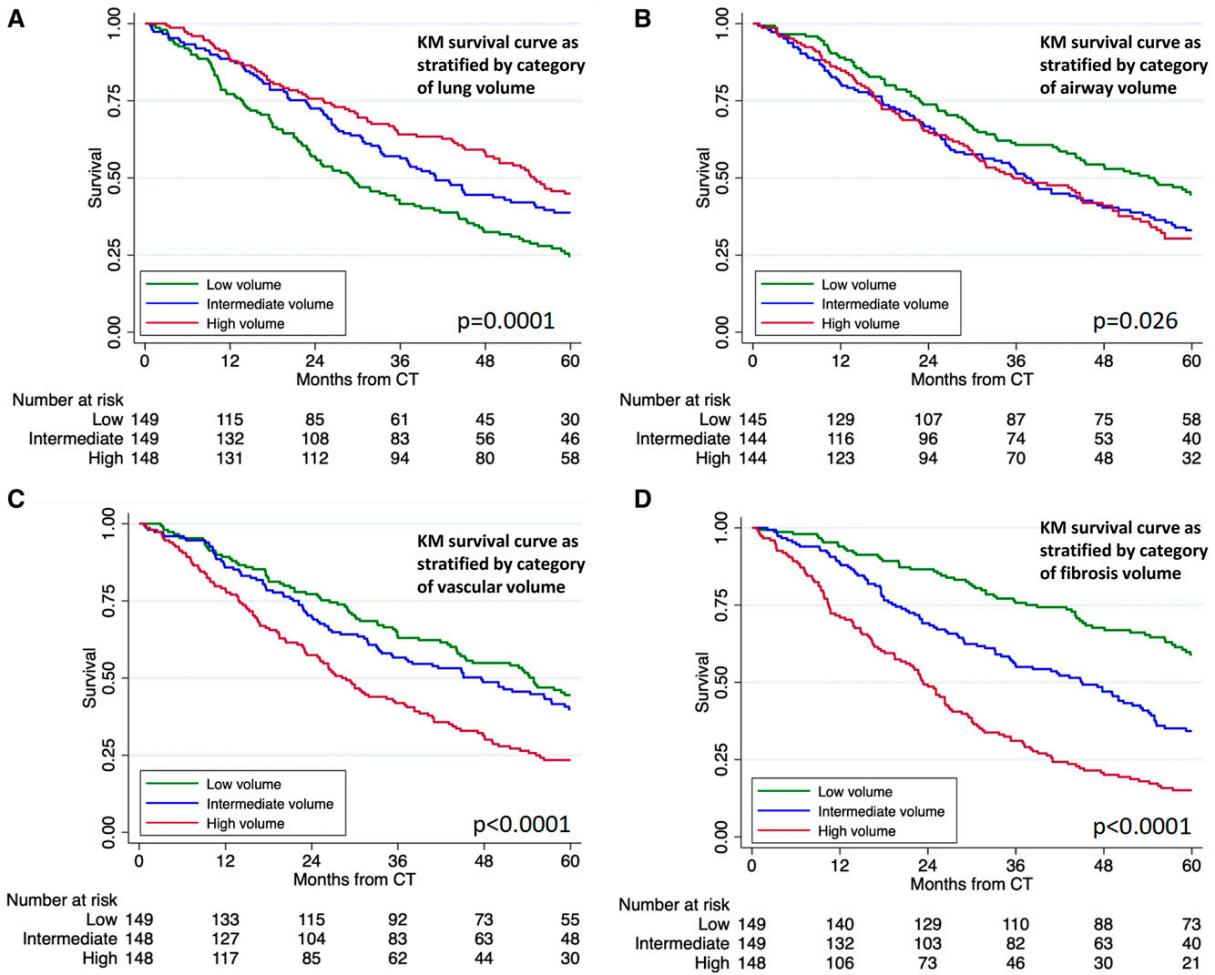
(*A–D*) KM survival curve for combined PROFILE cohort stratified by category (tertile) of lung volume (*A*), airway volume (*B*), vascular volume (*C*), and fibrosis volume (*D*). CT = computed tomography; KM = Kaplan-Meier; PROFILE = Prospective Observation of Fibrosis in the Lung Clinical Endpoints.

In cross-sectional analysis, baseline CT scans with lower lung volume, higher vascular volume, higher airway volume, and higher fibrosis volume were all associated with reduced five-year survival in both the discovery and validation cohorts (Table 2). When adjusted for baseline disease severity, lung volume (hazard ratio [HR], 0.98 [95% confidence interval (CI), 0.97–0.99]; *P* = 0.007), Vascular volume (HR, 1.37 [95% CI, 1.20–1.56]; *P* < 0.001), fibrosis volume (HR, 1.17 [95% CI, 1.12–1.22]; *P* < 0.001), and airway volume (HR, 5.41 [95% CI, 1.87–15.66]; *P* = 0.002) all maintained this survival association in the cohort as a whole. When stratifying by tertile, each measure effectively discriminated survival in combined cohort analysis (Figures 3A–3D). In the additional Cambridge validation set, lung volume, vascular volume, and fibrosis volume were all associated with five-year survival. When stratifying by category, lung volume and fibrosis volume both still discriminated survival (*see* Figure E1 in the online supplement).

**Table 2.** Unadjusted Associations between Baseline Quantitative CT Features and Five-Year Survival in the PROFILE and Cambridge Cohorts

CT Measure	PROFILE Discovery (*n* = 223)			PROFILE Validation (*n* = 223)			Cambridge Validation (*n* = 195)		
	HR	95% CI	*P* Value	HR	95% CI	*P* Value	HR	95% CI	*P* Value
Lung volume	0.98	0.96–0.99	0.001	0.97	0.96–0.99	0.003	0.98	0.96–0.99	0.005
Airway volume	1.90	0.46–7.82	0.374	10.09	2.06–49.35	0.004	1.82	0.96–3.45	0.068
Vascular volume	1.43	1.20–1.69	<0.001	1.31	1.09–1.59	0.005	1.23	1.01–1.50	0.042
Fibrosis volume	1.22	1.17–1.28	<0.001	1.22	1.15–1.30	<0.001	1.11	1.05–1.18	0.001

*Definition of abbreviations*: CI = confidence interval; CT = computed tomography; HR = hazard ratio; PROFILE = Prospective Observation of Fibrosis in the Lung Clinical Endpoints.

### Progression-Free Survival Analysis

The protocolized, prospective nature of PROFILE allowed us to assess near-term outcomes. Lower lung volume, increased vascular volume, and increased fibrosis volume were again associated with reduced progression-free survival in the discovery and validation cohorts. Lung volume (HR, 0.98 [95% CI, 0.96–0.99]; *P* = 0.001), vascular volume (HR, 1.30 [95% CI, 1.12–1.51]; *P* = 0.001), and fibrosis volume (HR, 1.17 [95% CI, 1.12–1.22]; *P* < 0.001) maintained this survival association in the cohort as a whole when the model was adjusted for baseline GAP index (Table 3) as well as through a sensitivity analysis iteratively adding each GAP component (*see* Table E1).

**Table 3.** Adjusted Association between Baseline Quantitative CT Features and Clinically Relevant Outcomes in the Pooled Prospective Observation of Fibrosis in the Lung Clinical Endpoints Cohort (*n* = 426)

CT Measure	*n*	Two-Year Progression-Free Survival			Five-Year Survival		
		HR	95% CI	*P* Value	HR	95% CI	*P* Value
Lung volume	427	0.98	0.96–0.99	0.001	0.98	0.97–0.99	0.007
Airway volume	415	1.45	0.43–4.85	0.545	3.74	1.28–10.92	0.016
Vascular volume	426	1.30	1.12–1.51	0.001	1.37	1.20–1.57	<0.001
Fibrosis volume	427	1.17	1.12–1.22	<0.001	1.17	1.12–1.22	<0.001

*Definition of abbreviations*: CI = confidence interval; CT = computed tomography; HR = hazard ratio.

Model adjusted for gender, age, and physiology stage at the time of CT. HRs are per 0.1-L change in high-resolution CT biomarker volume.

### Longitudinal Analysis

**Figure 4.**
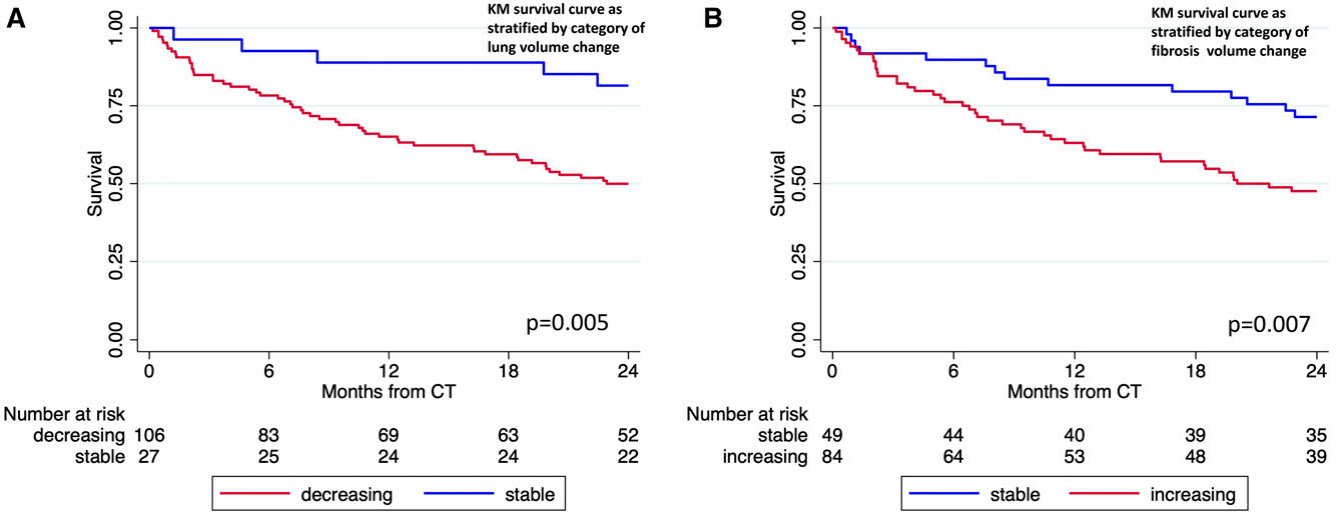
(*A* and *B*) KM survival curves for PROFILE participants who underwent serial CT stratified by categorical change in (*A*) lung volume and (*B*) fibrosis volume. For definition of abbreviations, *see* Figure 3.

In an exploratory longitudinal analysis of 134 patients who underwent repeat CT 6–24 months after baseline CT, lung volume decreased by 8.9% annually (95% CI, −14.38% to −3.43%; *P* = 0.001), and fibrosis volume increased by 13.2% (95% CI, 3.59% to 22.85%; *P* = 0.007). Vascular volume did not significantly change over time, and airway volume values could not be estimated, as model assumptions were not satisfied. When assessing survival after the last CT scan obtained, those with decreasing lung volume had a greater than threefold increased risk of death (HR, 3.41 [95% CI, 1.36–8.54]; *P* = 0.009) compared with those with stable or increasing lung volume (Figure 4A). Those with increasing fibrosis volume had more than twofold increased risk of death (HR, 2.23 [95% CI, 1.22–4.08]; *P* = 0.009) compared with those with stable or decreasing fibrosis volume (Figure 4B).

## Discussion

We demonstrate that CT scans from patients with IPF can be used to train and develop models that can rapidly segment CT scans at scale to produce data on fibrosis, vessel, airway, and lung volumes and that these can predict both progressive disease and mortality. All four of our CT imaging models at baseline are predictive of mortality. Fibrosis extent, vascular volume, and lung volume are also predictive of near-term progressive disease. The PROFILE dataset is unique in that it consists of patients who were treatment naive in that the data were collected before the advent of widespread antifibrotics. Nevertheless, to validate the models further, we applied them to another dataset of real-world patients from Cambridge. Although those patients were from a smaller dataset and had the confounding factor that many were treated with antifibrotics, baseline lung volume, vascular volume, and fibrosis volume in that additional cohort were all still associated with 5-year survival.

A longitudinal analysis of CT scans in patients with multiple CT scans showed that over time, there were significant reductions in lung volume and increases in fibrosis volume. This is consistent with our understanding of IPF disease progression, with progressive loss of volume and fibrosis extent. Although baseline vascular volume predicted mortality, it is interesting that we observed no significant change over time in longitudinal imaging. Our findings echo the data linking vessels (or vessel-related structures as defined by Computer-Aided Lung Informatics for Pathology Evaluation and Rating [CALIPER]) with mortality in IPF (18, 19). It may be that although other measures (such as fibrosis volume) change as IPF progresses, the vascular volume in the lung (while being predictive of mortality) is slower to change and is therefore a less useful marker longitudinally.

Exploring associations with the segmentation models and conventional markers of disease severity in IPF, the lung volume model showed a positive correlation with FVC. We previously used this model to show a correlation over time in an independent dataset of 93 patients with CT scans and lung function data over 7 years, in which we showed a significant difference in CT lung volume decline between progressive and stable cohorts, with annual progression defined by five variables: survival time and annualized changes in FVC, FEV_1_, Dl_CO_, and Va (20). It is interesting that none of the other models here were associated with either FVC or Dl_CO_, despite their being associated with progression (fibrosis and vessel models), mortality (fibrosis and vessel models), and change over time (fibrosis and lung volume models). This suggests that the fibrosis, vessel, and airway volume models may be measuring IPF disease severity signals that are independent of pulmonary function and may help identify subtypes of patients to study in more detail or may help explain differing treatment responses across large cohorts.

A number of visual scoring systems have been used to assess the severity of fibrotic lung disease on the basis of anatomical findings on CT (21, 22). These methods are labor intensive, subjective, and prone to poor interobserver variability. Although these visual scoring systems can be performed by radiologists, manual segmentation of individual CT scans cannot be performed at scale manually, as significant radiology expertise and time are required. The use of deep learning to segment CT scans from patients with IPF at scale is a novel approach that has a number of applications for the development of imaging-based biomarkers in this disease.

The advantages of this method (compared with both manual segmentation and a visual scoring system) include the speed of segmentation and the production of quantifiable data (e.g., volume of airways) compared with a scoring system that produces an aggregate of visual scores. Computer-assisted approaches to classify and quantify CT patterns have been available for nearly four decades (23, 24). CALIPER offers a computer-vision approach based on volumetric local histogram and morphologic analysis, which labels automatically the typical high-resolution CT (HRCT) parenchymal features (25). It also allows the quantification of pulmonary vascular volume, which has been shown to be a strong predictor of FVC decline in patients with IPF (26).

Another technique, quantitative lung fibrosis (QLF), uses a support vector machine to quantitatively measure honeycombing, ground-glass opacity, and a composite quantitative interstitial lung disease score (27). QLF and quantitative interstitial lung disease scores have been used as surrogate endpoints in seven IPF clinical trials to date, showing correlations with FVC change and treatment efficacy (28). An alternative method is functional respiratory imaging (FRI), in flow simulations are performed within the airways and pulmonary vessels are extracted from low-dose HRCT scans taken during inspiration and expiration (29). Changes in airway caliber using FRI have been shown to correlate with IPF disease progression (30).

The Systematic Objective Fibrotic Imaging Analysis Algorithm, a deep learning–based approach to classify usual interstitial pneumonia (UIP) on HRCT, has been shown to provide enhanced outcome prediction in patients with progressive fibrotic lung disease compared with expert radiologist evaluation or guideline-based histologic pattern (31). More recently, a larger study of more than 2,000 patients with IPF showed the development of a machine learning–based UIP classifier that classified patients as “UIP positive” if they had a significantly greater annual decline in FVC than those classified as “UIP negative” (32).

The segmentation approaches adopted in our models have a number of significant advantages and benefits over the previously described approaches. First, rather than arbitrary scoring systems, we produce quantifiable volume data for lung, airway, vessel, and fibrosis volume. The fibrosis and airway models have been trained and segmented from a variety of real-world CT scans from multiple (*n* = 5) imaging vendors and at various slice thicknesses between 0.5 and 5 mm. These models are therefore able to segmentate images from a wide range of CT scans in patients with IPF, unlike other methods that have rigid criteria for image suitability, such as CALIPER, which requires scans with <1-mm slice thickness with a reconstruction algorithm that is not too sharp (e.g., ⩽B70 on a Siemens scanner but not B80) (33). Our segmentation models are therefore likely to be more broadly clinically applicable.

The overall segmentation success rate across all four models on our platform was high, with almost all (97.8%) CT scans (which were all collected as part of routine clinical care) successfully segmented into the four models. This is higher than with other described computer-assisted classification approaches, which can be affected by a number of factors, including reconstruction algorithm, pixel size, and CT scanner used (34). We showed that in addition to a high rate of segmentation success, the Dice scores for our models did not differ according to any of the image attributes, such as scanner manufacturer, convolution kernel, and slice thickness. Furthermore, in comparison with techniques such as FRI that require specific protocols for patient inspiration to capture CT images (35), our models were applied to CT scans that were collected during the course of routine clinical care, with no prespecified patient breathing cycle or image specification protocol. Three of our models (fibrosis, vascular, and airway) were also created using three-dimensional convolutional neural networks (compared with two-dimensional slice models used in computer techniques such as QLF), which allows a detailed volume analysis from each CT scan.

Our work has several limitations. One is that the PROFILE CT data were collected as part of routine clinical care rather than as part of a protocolized clinical trial. However, this is also a strength, as the segmentations were performed on CT scans from multiple scanners, with multiple reconstructions and slice thicknesses, but still maintained a high segmentation success rate. The scans were obtained during inspiration but were not spirometry controlled; had they been, the correlation between lung volume and FVC might have been stronger. Finally because the data were collected as part of routine clinical care, there was limited synchronization between CT and PFT time, and for our analysis of CT model against FVC and Dl_CO_, we chose data that were within 180 days of each other. We did show a strong correlation between FVC and lung volume, if we had had data that were more contemporaneous, we might have seen other correlations between CT models and pulmonary function.

Segmentation models have a number of uses in the management of patients with IPF. In the clinic, they might be used to provide information on disease progression to help both patients and clinicians make decisions on the timing of antifibrotic initiation or transplantation. As multiple IPF therapies become available, these models could also be used as relatively short term prognostic biomarkers to inform early change of therapy because of nonresponse to medication. The prediction of mortality from baseline CT scans can also help patients with decisions regarding quality of life versus end-of-life care. In the clinical trial setting, apart from their obvious potential as novel endpoints, these models could be used to help stratify trial participants into studies and both allow researchers to select patients with disease that is more likely to progress (and therefore increase the possibility of finding a treatment effect between study arms) and ensure that study groups are as carefully matched as possible to ensure that any difference between arms is real.

### Conclusions

We have shown the ability to automate the segmentation and interpretation of CT scans, providing prognostic information on the basis of a baseline scan on both progression-free survival and mortality in patients with IPF. Longitudinal changes in radiological lung volumes, airways, and fibrosis extent can also then identify progressive disease. These segmentation algorithms could easily be integrated into routine clinical practice to help provide prognostic information to patients with this complicated and progressive lung disease, and they show promise as future key endpoints or imaging biomarkers in clinical trials.
